# Mapping the availability of translated versions of posttraumatic stress disorder screening questionnaires for adults: A scoping review

**DOI:** 10.1080/20008066.2022.2143019

**Published:** 2022-11-25

**Authors:** Joel Hoffman, Ziv Ben-Zion, Adrián Arévalo, Or Duek, Talya Greene, Brian J. Hall, Ilan Harpaz-Rotem, Belinda Liddell, Cosima Locher, Naser Morina, Angela Nickerson, Monique C. Pfaltz, Matthis Schick, Ulrich Schnyder, Soraya Seedat, Fatlinda Shatri, Hao Fong Sit, Roland von Känel, Tobias R. Spiller

**Affiliations:** a School of Psychology, UNSW Australia, Sydney, Australia; b Department of Psychiatry, Yale University School of Medicine, New Haven, CT, USA; c US Department of Veterans Affairs National Center for PTSD, VA Connecticut, Healthcare System, West Haven, CT, USA; d Facultad de Medicina & Neuron Research Group Lima, Universidad de Piura, Lima, Perú; e Facultad de Medicina “San Fernando”, Universidad Nacional Mayor de San Marcos, Lima, Perú; f Department of Community Mental Health, University of Haifa, Haifa, Israel; g Center for Global Health equity, New York University (Shanghai), Shanghai, People’s Republic of China; h School of Global Public Health, New York University, New York, NY, USA; i Department of Consultation-Liaison Psychiatry and Psychosomatic Medicine, University Hospital Zurich, University of Zurich, Zurich, Switzerland; j Department of Psychology and Social Work, Mid Sweden University, Östersund, Sweden; k University of Zurich, Zurich, Switzerland; l Department of Psychiatry, Faculty of Health Sciences, Stellenbosch University, Cape Town, South Africa; m Department of Psychology, Faculty of Social Sciences, The University of Hong Kong, Hong Kong, People’s Republic of China

**Keywords:** Scoping review, PTSD, protocol, registered report, screening, questionnaire, translation, Revisión exploratoria, TEPT, protocolo, informe registrado, tamizaje, cuestionario, traducción, 范围界定综述, PTSD, 方案, 注册报告, 筛选, 问卷, 翻译

## Abstract

**Background:** The most used questionnaires for PTSD screening in adults were developed in English. Although many of these questionnaires were translated into other languages, the procedures used to translate them and to evaluate their reliability and validity have not been consistently documented. This comprehensive scoping review aimed to compile the currently available translated and evaluated questionnaires used for PTSD screening, and highlight important gaps in the literature.

**Objective:** This review aimed to map the availability of translated and evaluated screening questionnaires for posttraumatic stress disorder (PTSD) for adults.

**Methods:** All peer-reviewed studies in which a PTSD screening questionnaire for adults was translated, and which reported at least one result of a qualitative and /or quantitative evaluation procedure were included. The literature was searched using Embase, MEDLINE, and APA PsycInfo, citation searches and contributions from study team members. There were no restrictions regarding the target languages of the translations. Data on the translation procedure, the qualitative evaluation, the quantitative evaluation (dimensionality of the questionnaire, reliability, and performance), and open access were extracted.

**Results:** A total of 866 studies were screened, of which 126 were included. Collectively, 128 translations of 12 different questionnaires were found. Out of these, 105 (83.3%) studies used a forward and backward translation procedure, 120 (95.2%) assessed the reliability of the translated questionnaire, 60 (47.6%) the dimensionality, 49 (38.9%) the performance, and 42 (33.3%) used qualitative evaluation procedures. Thirty-four questionnaires (27.0%) were either freely available or accessible on request.

**Conclusions:** The analyses conducted and the description of the methods and results varied substantially, making a quality assessment impractical. Translations into languages spoken in middle- or low-income countries were underrepresented. In addition, only a small proportion of all translated questionnaires were available. Given the need for freely accessible translations, an online repository was developed.

**HIGHLIGHTS**

- We mapped the availability of translated PTSD screening questionnaires.
- The quality of the translation and validation processes is very heterogenous.
- We created a repository for translated, validated PTSD screening questionnaires.

## Abstract (es)

**Antecedentes**: Los cuestionarios más usados para el tamizaje del TEPT en adultos fueron desarrollados en inglés. Aunque muchos de esos cuestionarios fueron traducidos a otros idiomas, los procedimientos usados para traducirlos y para evaluar su confiabilidad y validez no han sido documentados consistentemente. Esta revisión exploratoria general buscó compilar los cuestionarios traducidos y evaluados actualmente disponibles para el tamizaje del TEPT y resaltar brechas importantes en la literatura.

**Objetivo**: Esta revisión tuvo por objetivo mapear la disponibilidad de cuestionarios de tamizaje traducidos y evaluados para el trastorno de estrés postraumático (TEPT) en adultos.

**Métodos**: La literatura fue buscada utilizando Embase, MEDLINE y APA PsycInfo, búsqueda de citaciones y contribuciones de los miembros del equipo de estudio. No hubo restricciones respecto al idioma objetivo de las traducciones. Se extrajeron datos respecto al procedimiento de traducción, la evaluación cualitativa, la evaluación cuantitativa (dimensionalidad del cuestionario, confiabilidad y rendimiento) y acceso abierto. Todos los estudios revisados por pares en los cuales un cuestionario de tamizaje del TEPT para adultos fue traducido, y que reportaron al menos un resultado de un procedimiento de evaluación cualitativo y/o cuantitativo.

**Resultados:** Se evaluó un total de 866 estudios, de los cuales 126 fueron incluidos. En forma colectiva, se encontró 128 traducciones de 12 diferentes cuestionarios. De estos, 105 estudios (83.3%) utilizaron un procedimiento de traducción hacia adelante y hacia atrás, 120 (95.2%) evaluaron la confiabilidad del cuestionario traducido, 60 (47.6%) la dimensionalidad, 49 (38.9%) el rendimiento, y 42 (33.3%) utilizaron procedimientos de evaluación cualitativos. Treinta y cuatro cuestionarios (27.0%) estaban disponibles libremente o accesibles bajo petición.

**Conclusiones**: Los análisis conducidos y la descripción de los métodos y resultados varió sustancialmente, haciendo impráctica una evaluación de calidad. Las traducciones a idiomas hablados en países de ingresos medios o bajos estuvieron sub-representadas. Adicionalmente, sólo una pequeña proporción de todos los cuestionarios traducidos estaba disponible. Dada la necesidad de traducciones disponibles libremente, se desarrolló un repositorio en línea.

## Abstract (zh)

**目的：**本综述旨在确定成人创伤后应激障碍 (PTSD) 的翻译和评估筛查问卷的可用性。

**简介：**最常用的成人 PTSD 筛查问卷是用英语开发的。尽管其中许多问卷被翻译成其他语言，但用于翻译和评估其可靠性和有效性的程序并没有得到一致的记录。这项全面的范围界定综述旨在汇编目前可用的 PTSD 筛查翻译和评估问卷，并突出文献中的重要不足。

**纳入标准：**所有翻译了成人 PTSD 筛查问卷，并报告了至少一项定性和/或定量评估程序结果的同行评议研究。

**方法：**使用 Embase、MEDLINE 和 APA PsycInfo、引文搜索和研究团队成员的贡献对文献进行搜索。翻译的目标语言没有限制。提取翻译程序、定性评价、定量评价（问卷的维度、可靠性和表现）和开放获取的数据。

**结果：**共筛选出866项研究，纳入了126项。总共找到了 12 份不同问卷的 128 份翻译。其中，105 项 (83.3%) 研究使用正向和反向翻译程序，120 项 (95.2%) 评估了翻译问卷的可靠性，60 项 (47.6%)含维度，49 项 (38.9%) 含表现，42 (33.3%）使用定性评估程序。 34 份问卷 (27.0%) 可免费获得或应要求提供。

**讨论：**所进行的分析以及对方法和结果的描述差异很大，使得质量评估不可实操。中等或低收入国家使用的语言翻译版本不足。此外，只有一小部分翻译后的问卷可用。鉴于需要免费获取翻译，开发了一个在线存储库。

## 1. Introduction

### 1.1. Background

Screening for symptoms of posttraumatic stress disorder (PTSD) is essential when working with trauma-exposed individuals in clinical as well as research settings. Most commonly, screening is undertaken with the help of a questionnaire either completed by or administered to the individual who is being assessed. In clinical settings, these screening measures are often used to identify those likely to meet a PTSD diagnosis, while in research settings, they are used in a variety of study designs for example in epidemiological studies to assess the prevalence of PTSD in populations (Schlenger et al., 2002; Terhakopian et al., 2008).

The purpose of screening measures is to be a brief, easy to administer instrument which reliably distinguish between individuals with and without PTSD. However, a screening measure is not designed to obtain a definitive, but only a probable diagnosis, which needs to be clinically verified. Therefore, screening measures can but do not have to comprise items corresponding to the diagnostic criteria of PTSD. In addition, they could include any item, which is predictive of a probable PTSD diagnosis and can effectively be assessed in the target scenario, in which the screening measure is assumed to be applied (for more details see Brewin, 2005). Over the last few decades, a multitude of PTSD screening questionnaires have been developed (Brewin 2005). Differences between these questionnaires include differences in diagnostic criteria sets for PTSD (e.g. as defined by the fourth or the fifth edition of the Diagnostic and Statistical Manual of Mental Disorders; DSM-IV, DSM-5; [O‘Donnell et al., 2014] or eleventh edition of the International Classification of Diseases; ICD-11), the number of items included (e.g. assessing all symptoms of PTSD (Blanchard et al., 1996) vs only a few symptoms (Connor & Davidson, 2001), different target populations (e.g. refugees [Mollica et al., 1992] or veterans [Yarvis et al., 2012]), different settings (e.g. clinic [Duek et al., 2020], or community [Kilpatrick et al., 2013]) and constructs assessed (e.g. only symptoms of PTSD [Foa et al., 1997] vs broader sequelae of trauma [Oe et al., 2020]). Many of these measures were developed in English and in high-income countries, most commonly in the United States of America. Psychological trauma, however, affects individuals all around the globe, including many who do not live in high-income countries and are not native English speakers (Benjet et al., 2016). Therefore, screening for symptoms of PTSD in these diverse settings requires either new, context-specific screening measures or translations of existing questionnaires (Beaton et al., 2000; Bullinger et al., 1998)]. In both cases, the ability of the questionnaire to reliably measure symptoms of PTSD in the specific context is essential for ensuring the clinical utility and validity of the research in which this questionnaire is utilized. Otherwise, if the measures are inadequate, this risks the misdiagnosis of PTSD, the inaccurate assessment of symptom severity, biased estimates of prevalence rates, compromised comparisons across regions or ethnic groups and unreliable results for use in meta-analyses (van de Vijver and Tanzer 2004). Furthermore, in clinical settings, unreliable screening tools may result in individuals affected by PTSD not receiving adequate treatment.

The translation of an existing questionnaire is an expensive, and time-consuming process. Historically, such translations have unfortunately often been handled ad hoc and were poorly documented and often have been inadequately evaluated (Chassany et al., 2002). Over the last decade, multiple guidelines, frameworks, and best-practice summaries that can guide the translation of questionnaires have been developed (Acquadro et al., 2008). Across all different guidelines, it is emphasized that a mere word-to-word translation of a questionnaire is insufficient to ensure reliability and validity in a target population and that cultural aspects of the target population also need to be taken into account, especially given that the trans-cultural validity of PTSD itself is a subject of ongoing debate (Hinton and Lewis-Fernández 2011; Gilmoor et al., 2019; Hall 2020). Yet, the recommended procedures differ substantially and indicate a lack of consensus regarding the procedures required to utilize a reliable translation.

Given the importance of PTSD screening questionnaires for clinicians, public health experts and researchers globally, free access to translated, accurate and reliable questionnaires is essential. Several literature reviews have discussed the availability of PTSD screening questionnaires beyond a US context (Ali et al., 2016; Beidas et al., 2015; Gagnon and Tuck (2004). Beidas and colleagues (2015) systematically reviewed the literature for freely available, validated, standardized screening questionnaires for mental disorders for adults in low-resource settings (Beidas et al., 2015). Their review also included five questionnaires assessing symptoms of PTSD in English (Beidas et al., 2015). However, they did not assess whether these questionnaires had been translated from English into other languages. In another systematic review, Ali et al., 2016) provided an overview of brief screening tools for the detection of common mental disorders, including PTSD, that were validated in low- and middle-income countries (Ali et al., 2016). Importantly, they aimed to assess the diagnostic accuracy against a recognized gold standard diagnostic interview. Regarding PTSD screening questionnaires, 13 studies investigating a total of 10 different questionnaires were identified, with some studies assessing the accuracy of a translated version, while others assessed the accuracy of the original, English version. Evaluating the diagnostic accuracy of translated screening questionnaires is not commonly done as part of the translation process. Hence, the review likely excluded many studies reporting on the translation of a PTSD screening questionnaire. In a third systematic review, Gagnon and Tuck (2004) aimed to determine the best questionnaires for assessing multiple outcomes, including PTSD, in refugee women (Gagnon and Tuck 2004). The study reported on the properties of seven different scales. Of these, the highest number of validation studies, namely 14, were reported for the Harvard Trauma Questionnaire (HTQ; Mollica et al., 1992). However, their review was limited to a specific subpopulation, namely refugee women.

Notwithstanding these reviews, a comprehensive overview of the available translated PTSD screening questionnaires for different populations is currently lacking. This is problematic for two main reasons. First, without an overview, gaps in the literature are difficult to address. Notably, a lack of translated questionnaires constitutes an important hurdle for clinicians and researchers. Furthermore, it seems likely that PTSD screening questionnaires are less available for non-English speaking populations in low- and middle-income countries than in high-income countries. This is particularly a disadvantage for trauma-affected individuals, clinicians, and researchers who do not live in high-income countries, creating further barriers to research and treatment in these contexts. Second, systematic investigations (e.g. such as a systematic review) prerequire enough available information to ensure that the research question of interest can be addressed with the current literature. Regarding translations of PTSD questionnaires, no overview of the relevant literature exists. In addition, the above outlined reviews identified few studies investigating the performance of a translated PTSD questionnaire (Ali et al., 2016) or had to forgo the reliability and validity assessment due to very heterogeneous outcome reporting (Beidas et al., 2015). Hence, the information to plan a systematic review which would answer a specific quality-related question is currently lacking and not the goal of this review.

We aim to provide a broad overview of the existing literature of PTSD screening questionnaires translated from English to other languages, as well as the evaluation process of these translations. Therefore, neither a quality appraisal of the existing translated questionnaires, nor recommendations regarding the use of specific questionnaires are goals of this *scoping review* (Arksey & O‘Malley, 2005; Nyanchoka et al., 2019; Peters et al., 2020).

### 1.2. Review question

The objective of this scoping review is to map the global availability of evaluated non-English versions of PTSD screening questionnaires for adult populations. Our definition of a non-English version is one that has been translated from English to another language independent of whether additional steps were undertaken to adapt and evaluate a questionnaire to a target population or not. Because we aim to map the available translations of PTSD screening questionnaires and expect that few translations have undergone a formal evaluation procedure, we define evaluated questionnaires as questionnaires on which any kind of information about their translation technique as well as their accuracy is provided in a peer-reviewed journal article. Therefore, some of the included questionnaires might not be evaluated in a formal sense (e.g. performance might be only assessed by correlating the total score to another PTSD questionnaire’s total score). More information about the conducted evaluation procedures will be provided for each study (see below). The aims of this review are twofold: First, to identify published, peer-reviewed PTSD screening questionnaires that were translated to at least two different languages. The decision to only cover peer-reviewed, published studies as well as to exclude questionnaires with less than two translations, was made to limit the scope of this review, to ensure its feasibility and to include a criterion reflecting the use of a given questionnaires across a minimal number of different language groups. The second aim is to map all evaluated translations of the screening questionnaires identified in step one in terms of translation techniques used, results from the qualitative and/or quantitative evaluations, and accessibility of the translated questionnaires.

## 2. Methods

### 2.1. Study design

The aim of a scoping review is to map existing evidence on a prespecified topic. In contrast to systematic reviews, scoping reviews are more exploratory and cover a broader spectrum of evidence (Peters et al., 2020). This scoping review will be conducted in accordance with the methodological framework for scoping reviews proposed by the Joanna Briggs Institute (Peters et al., 2015; Peters et al., 2020). This framework is based upon earlier work by Arksey and O‘Malley (2005) and Levac and colleagues (Levac et al., 2010). The reporting will follow the guidance of the extension of the ‘Preferred Reporting Items for Systematic Reviews and Meta-Analyses’ for scoping reviews (PRISMA-ScR; Tricco et al., 2018).

### 2.2. Consultation

Several actions were taken to ensure adequate consultation from relevant stakeholders. First, the study team was assembled specifically to include researchers with a diverse background regarding gender, profession, cultural context, and geographical location. Second, all members of the research team will consult with local and international contacts (personal and professional). Third, the project was designed to be published as a registered report. Registered reports follow a two-phase publishing model (Nosek and Lakens 2014; Nosek et al., 2018). In a first phase, the authors only submit documents outlining the importance of the question they want to investigate and a detailed study plan including information about the proposed methodological approach and study design. These documents will then be reviewed by independent peers who can suggest modifications to all aspects of the submitted documents (including the study design, the measures etc.). If the study is deemed to investigate a relevant question in an appropriate manner, an ‘in-principle acceptance’ is granted meaning that the final manuscript (including the results and their discussion) will be published after the completion of the study, regardless of what the results show (Nosek et al., 2018; Simons et al., 2014). Due to this publishing model, independent peer-reviewers, will be consulted before the review will be conducted.

### 2.3. Review registration

The initial protocol including the appendices were revised upon the suggestions made by the reviewers during the first-phase review process. Following journal guidelines on registered reports, after the revised manuscript and the protocol were accepted ‘in-principle,’ the revised protocol was deposited and time-stamped here: https://osf.io/ud8hc. We used the Open Science Framework (OSF; https://osf.io), an open-source platform maintained by the Center for Open Science, a non-profit technology organization.

### 2.4. Eligibility criteria

**Figure 1.**
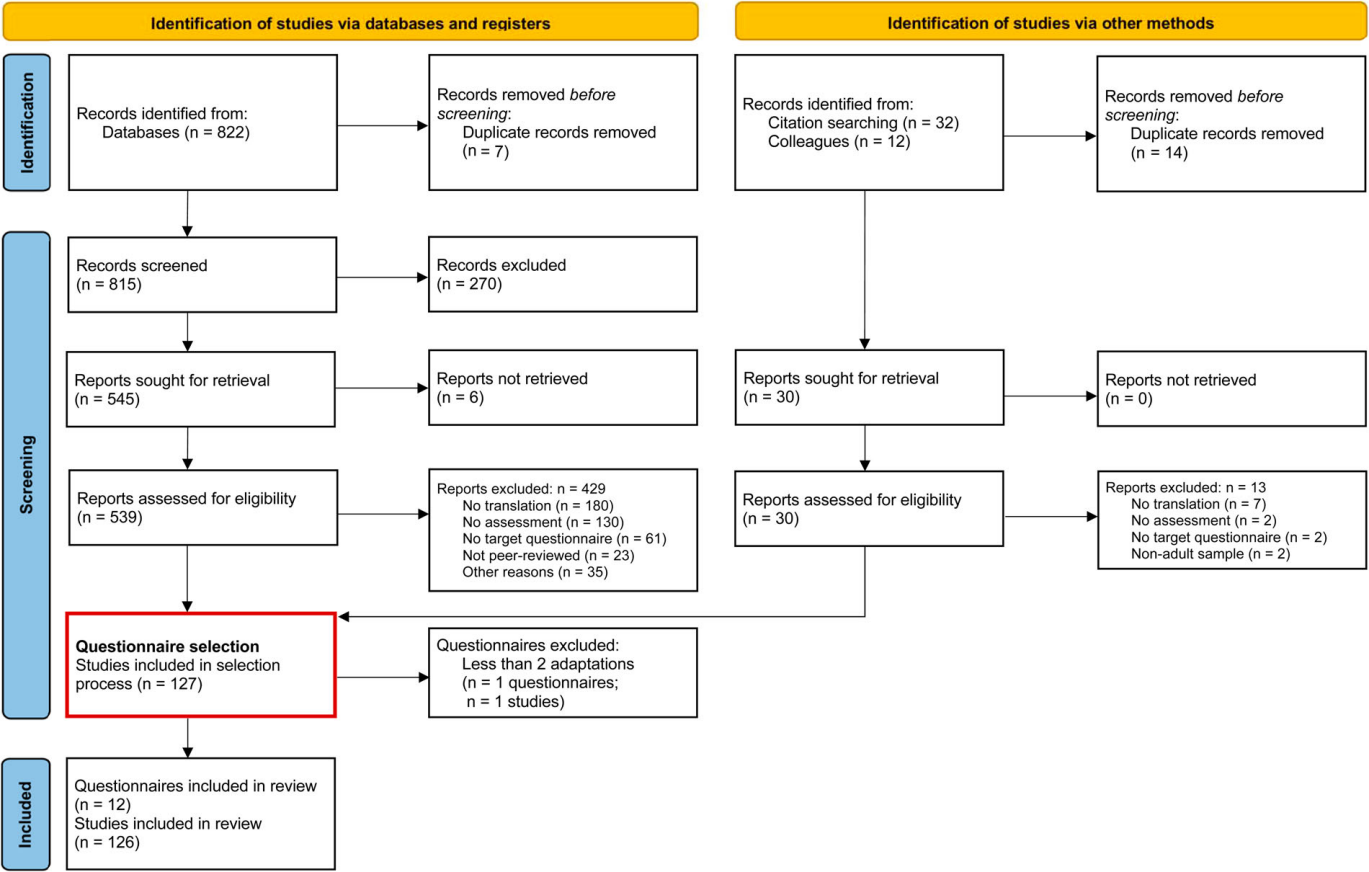
Flow of information through different phases of the review, detailing in- and excluded papers.

To ensure the feasibility of this review, its scope will be limited to PTSD screening questionnaires that were translated at least two different times. Two translations may include translations into different language (e.g. French, Arabic, Spanish), different versions or dialects within a given language (e.g. Levantine Arabic, Egyptian Arabic, Modern Standard Arabic), and different translations within the same language and dialect (e.g. Somali translations that differ based on translation team). Therefore, the inclusion of records is conducted in a two-step process which is shown in Figure 1 (which is an adaption of the PRISMA flow diagram; Page et al., 2021). In a first step, we will systematically search for peer-reviewed articles containing information on the translation of PTSD screening questionnaires for adults. A list with all translations for each questionnaire will be prepared. In a second step, all questionnaires for which less than two translations have been identified, will be removed prior to the data extraction. For all questionnaires that were adapted to account for revised PTSD diagnostic criteria (e.g. with one version assessing DSM-IV and a later version assessing DSM-5 criteria), the cumulative number of adaptations (across all versions of the questionnaires) will be assessed. If this cumulative number is five or higher, all versions of the questionnaires will be included in the data extraction process. The eligibility criteria for both steps are defined following the *Population, Concept, Context* (PCC) framework as follows:

**Populations:** This review focuses on translations of screening questionnaires developed for adult populations (≥ 18 years). No additional restrictions are imposed for the inclusion of populations (e.g. no restrictions regarding language, gender, country of origin, study site, whether the population was an epidemiological or clinical sample etc.).

**Concept:** The overarching concept of interest for this review is to map the availability of translated and evaluated PTSD screening questionnaires commonly used in traumatic stress research and/or in clinical practice. The following aspects of the concept of interest are discussed in depth: (a) PTSD, (b) screening questionnaire, (c) translation and/or cultural adaptation (d) evaluation.

*PTSD:* For the purpose of this review, questionnaires developed to assess PTSD as defined by either the fourth, the text-revised fourth or the fifth edition of the Diagnostic and Statistical Manual of Mental Disorders (DSM-IV, DSM-IV-TR, DSM-5; American Psychiatric Association, 2013) or the tenth or eleventh edition of the International Classification of Diseases (ICD-10, ICD-11; World Health Organization, 1992) are considered.

*Screening questionnaire*: In accordance with the systematic review of PTSD screening instruments for adults by Brewin, screening questionnaires were defined as consisting of less than 30 items (not including the items assessing the types of trauma experienced or witnessed (Brewin, 2005). A list outlining the questionnaires that will be included in the first step is provided in Appendix I (Supplemental data).

*Translation*: The translation of a questionnaire to a new population is a resource-intensive and complex process (Sousa & Rojjanasrirat, 2011). For this review, we consider all studies that include at least one translation of a PTSD screening questionnaire from English to any other language. Each new translation, independent from the number of preexisting translations into the same target language, will be counted as one translation. In contrast, studies that adapted a questionnaire to a population without translating it will be excluded from this review.

*Evaluation*: The translation process of a questionnaire should include an evaluation of the questionnaire’s properties, including its reliability and validity (Boateng et al., 2018). For this review, only studies that report information about the translation procedure as well as results from a performance assessment are considered for inclusion. Consequently, articles that state that a questionnaire was translated, but do not report some results information regarding what translated questionnaires exist as well as the evaluation procedure they have undergone and not to answer a specific question (e.g. regarding the quality the included translations). Therefore, we will collect information about the evaluation of the translated questionnaires (e.g. whether the reliability of the questionnaire was assessed) but *not* the quality of the included studies (e.g. the reported sensitivity and specificity). Consequently, to cover the presumably very heterogeneous literature, we will include studies with any kind of information on the questionnaire’s performance, even if this information was gained using non-standard practices.

**Context:** We will consider studies with no restrictions regarding the context in which they were conducted.

**Types of evidence sources:** Only peer-reviewed, published studies reporting quantitative and qualitative results of the evaluation of the adapted questionnaire will be considered. Such studies can include but are not limited to the following designs: epidemiological study, experimental study, and cross-sectional studies. Qualitative studies and any kind of study not reporting quantitative or qualitative results of the evaluation of the adapted questionnaire(s) will not be considered for this review.

**Languages:** Articles published in English, German, Spanish, French, Chinese, Filipino and Hebrew will be included.

**Date range.** The publication date range will not be limited. However, the definitions of PTSD considered for this review are limited to the DSM-IV, DSM-IV-TR, DSM-5, ICD-10, and ICD-11, of which the DSM-IV was introduced in 1994 and the ICD-10 in 1990.

### 2.5. Search strategy

Our search strategy for the identification of articles of interest rests upon four main pillars to ensure coverage of the index, but also of the grey literature. First, we will conduct a systematic search of three electronic bibliographic databases: Embase, MEDLINE, and APA PsycInfo. Second, we will search the reference list of relevant review articles (Ali et al., 2016; Beidas et al., 2015; Gagnon and Tuck 2004). Third, all members of our research team, of whom some have several decades of experience translating and adapting questionnaires, will be asked to provide articles of interest from their personal archives. Fourth, we will search PTSDpubs (formerly PILOTS), a curated database covering literature on PTSD.

The search strategy for the bibliographic databases was developed in several steps. Initially, a limited search of MEDLINE was conducted to identify relevant articles. Index terms and relevant words from the identified articles’ titles and abstracts were obtained. Next, the search strategies of existing systematic reviews (Beidas et al., 2015; Boateng et al., 2018; Gagnon and Tuck 2004) were analysed. Subsequently, a search strategy was developed by three authors (JH, FS & TS), and reviewed by the remaining authors as well as by an experienced librarian. We will search Embase, MEDLINE, and APA PsycInfo; using OVID. The final search strategy is presented in Appendix II (Supplemental data).

### 2.6. Source of evidence selection

Following the search, all citations identified through each pillar of the search strategy will be exported into *Covidence* (Veritas Health Innovation 2022). First, duplicates will be removed. Second, titles and abstracts of all remaining citations will be screened by a pair of independent reviewers against the inclusion criteria. The list with inclusion and exclusion criteria used by the reviewers is presented in Appendix III (Supplemental data). All abstracts deemed to meet inclusion criteria by at least one reviewer will be included in the review. Third, potentially relevant records will be fully retrieved. Again, a pair of reviewers will independently assess the records against the inclusion criteria. All articles deemed to meet inclusion criteria by at least one reviewer will be included in the review. Following the PRISMA-ScR guidelines (Tricco et al., 2018), the results of the search, the records inclusion process, and reasons for the exclusion of records during the full-text review will be recorded and reported. At the beginning of the evidence selection process, 25 randomly sampled records will be selected, and each will be screened by all reviewers. Afterwards, the results will be compared, and disagreements discussed. Based on this discussion, the document providing clarifications to the inclusion and exclusion criteria (Appendix III, Supplemental data) will be revised. All articles deemed to meet inclusion criteria by at least one reviewer will be included in the review.

### 2.7. Data extraction

The data extraction process will be guided by a data extraction form and conducted by a pair of reviewers using *Covidence*, adhering to the JBI Manual for Evidence (Peters et al., 2020). Any disagreements between the two reviewers during data extraction will be resolved by a third reviewer. If more than a total number of 30 studies are identified, data extraction will be conducted by one reviewer only to ensure feasibility of the review. In this case, accuracy of the extracted data they will be verified by an additional reviewer. Any disagreements will be resolved by a third reviewer.

### 2.8. Data of interest and data extraction form

The data of interest aimed to be extracted from individual records can be grouped into three categories: (a) Study characteristics, (b) Translation and evaluation, and (c) Access.

*Study characteristics*: For each record, the following data will be assessed:

**Table:** 

I.	Study name
II.	DOI
III.	Corresponding author’s email address
IV.	Year of publication
V.	Source questionnaire
VI.	Target population (Options: civilian, post-conflict/veteran, forcibly displaced)
VII.	Target setting (Options: clinical, emergency, community)
VIII.	Target language
IX.	Country of study site
X.	Sample size
XI.	Percentage of females

*Translation and evaluation:* As outlined above, multiple recommendations and guidelines for the translation of existing questionnaires exist (Acquadro et al., 2008). Still, very few studies have tested how different translation processes affect the validity of the corresponding translations (e.g. Perneger et al., 1999). Therefore, we only aim to map whether some basic steps regarding the translation procedures and performance testing of the resulting questionnaires were undertaken. For this purpose, we use the following three dimensions:

*Translation.*

**Table:** 

Was the measure back translated?
Grading: Yes or no.
Description: Kind of translation procedure used (free text).
I. Qualitative evaluation
Was any kind of qualitative validation process reported (e.g. pilot testing of the adapted questionnaire, qualitative interviews, face validity etc.)?
Grading: Any or none.
Description: Kind of qualitative assessment (free text).
II. Quantitative evaluation/Dimensionality
Was any kind of dimensionality testing conducted (e.g. confirmatory factor analysis, bifactor modelling, measurement invariance testing, etc.)?
Grading: Any or none.
Description: Kind of dimensionality assessment (free text).
III. Quantitative evaluation/Reliability
Was any kind of reliability testing reported (e.g. internal consistency, test-retest reliability etc.)?
Grading: Any or none.
Description: Kind of reliability assessment (free text).
IV. Quantitative evaluation/Performance
Was any kind of performance testing reported (e.g. diagnostic accuracy, correlation with another screening questionnaire, etc.)?
Grading: Any or none.
Description: Kind of performance assessment (free text).

*Access.* We will assess whether the source and the adapted questionnaires are freely available for clinical or research purposes. A questionnaire will be considered freely accessible, when it can either be freely accessed on the internet with a reasonable amount of effort (e.g. questionnaires only included in the supplementary materials of paywalled articles are not considered freely accessible) or can be requested from the authors with responses including the questionnaire within four weeks (also see Appendix V, Supplemental data).

**Table:** 

I.	Is the adapted questionnaire freely accessible?
II.	Grading: Yes or no.
III.	Description: Kind of access (free text).

A draft of the extraction form is provided in Appendix IV (Supplemental data). The data extraction form will be implemented with instructions into Covidence. The data extraction process will be piloted using five randomly selected full-text records which will be assessed by the involved data extraction reviewers. Disagreements will be discussed, and the extraction form will be revised as necessary with any resulting modifications explicitly stated in the resulting scoping review.

### 2.9. Deviations from the protocol

We report one deviation from the protocol. The search terms outlined in the protocol to be used in Embase and APA PsycInfo were missing an additional specifier included in the search terms for MEDLINE. Thus, we expanded the preregistered search terms (instead of ‘exp posttraumatic stress disorder’ they also included ‘or (ptsd or posttraumatic stress or post traumatic stress or trauma or traumatic) mp.’) for these two databases to increase consistency across all searched databases. However, this deviation only broadened the search criteria and did therefore not jeopardize the preregister aim of this scoping review.

## 3. Results

### 3.1. Study selection

The flow through the study selection process is shown in Figure 1. We identified 822 studies using our search strategy. An additional 32 studies were found by citation searching, and 12 were sent to us by colleagues. Thereof, 740 studies were excluded during the screening process, leaving 126 studies for extraction. The full screening details, including causes for exclusion, are shown in Figure 1. Interrater reliability for each of the three pairs of reviewers who reviewed titles, abstract, and fulltexts was between fair and substantial (Cohen’s κ 0.28, 0.58, and 0.61, respectively).

### 3.2. Study characteristics

**Figure 2.**
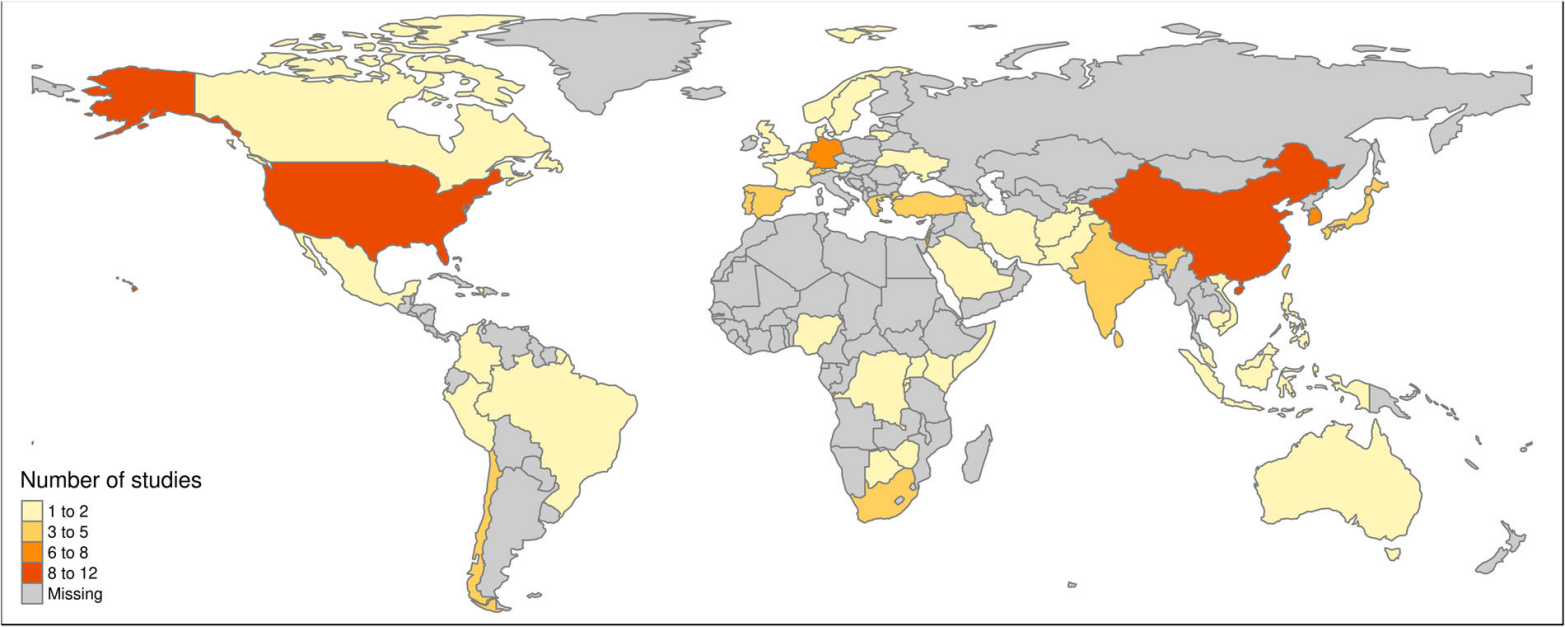
Map of study locations.

The details of the included studies are outlined in Table 1. Two of the 126 studies evaluated 2 questionnaires, so that from a total of 128 source questionnaires, 12 different PTSD screening measures were included, namely: The PTSD Checklist (PCL; *n* = 23; 18.0%); the Harvard Trauma Questionnaire (HTQ; *n* = 21; 16.4%); the Impact of Event Scale-Revised (IES-R; *n* = 21; 16.4%); the PTSD Checklist for the DSM-5 (PCL-5; *n* = 15; 11.7%); the International Trauma Questionnaire (ITQ; *n* = 14; 10.9%); the Posttraumatic Diagnostic Scale (PDS; *n* = 13; 10.2%); the Davidson Trauma Scale (DTS; *n* = 7; 5.5%); the Global Psychotrauma Screen (GPS; *n* = 3; 2.3%); the PTSD Symptom Scale-Interview (PSS-I; *n* = 3; 2.3%); the Harvard Trauma Questionnaire for DSM-5 (HTQ-5; *n* = 2; 1.6%); the Posttraumatic Diagnostic Scale-5 (PDS-5; *n* = 2; 1.6%); the Primary Care PTSD Screen (PC-PTSD: *n* = 2; 1.6%); and the Trauma Screening Questionnaire (TSQ; *n* = 2; 1.6%). Most studies evaluated the translations in civilian populations (*n* = 89; 70.6%), followed by forcibly displaced (*n* = 25; 19.8%), post-conflict/veteran (*n* = 10; 7.9%), and mixed (*n* = 3; 2.4%) populations. Study settings were clinical (*n* = 38; 30.2%), community (*n* = 74; 58.7%), emergency departments (*n* = 5; 4.0%), and mixed settings (*n* = 10; 7.9%). Sample sizes ranged from 18 to 7034 participants. Countries in which studies were conducted can be seen in Figure 2. The most common target languages were Arabic (*n* = 16), Spanish (*n* = 14), Chinese (*n* = 8), German (*n* = 7), Korean (*n* = 5), and French (*n* = 5); with all other languages ranging from 2–4 translations.

**Table 1.** Study characteristics.

Authors	Questionnaire	Target language	Target population	Target setting	Country of study site	Sample size (*N*)	Female (%)	Translation	Evaluation				Access
									Qualitative	Dimensionality	Reliability	Performance	
Bobes et al. (2000)	DTS	Spanish	Civilian	Clinical	Spain	86	65.12				X	X	
Chen et al. (2001)	DTS	Chinese	Civilian	Clinical	Taiwan	210	60.5	F & B		X	X	X	
Karanikola et al. (2021)	DTS	Greek-Cypriot	Civilian	Clinical	Cyprus	69	32.9	F & B	X	X	X	X	
Kontoangelos et al. (2017)	DTS	Greek	Civilian	Clinical	Greece	294	40.8	F & B		X	X		
Leiva-Bianchi & Araneda (2013)	DTS	Spanish	Civilian	Comm	Chile	291	unclear			X	X	X	
Morales Miranda (2006)	DTS	Spanish	Civilian	Comm	Peru	100	68			X	X		
Seo et al. (2008)	DTS	Korean	Civilian	Clinical	Korea	S1:93 S2:73 S3:88	S1:51.6 S2:38 S3:56.8	F & B		X	X	X	
Oe et al. (2020)	GPS	Japanese	Civilian	Clinical	Japan	58	93	F & B	X		X	X	Free online
Olff et al. (2020)	GPS	Arabic German Indonesian	Mixed	Mixed	Netherlands Germany Indonesia	S1:84 S2:40 S3:129	S1:56 S2:87.5 S3:60.5	F & B	X		X	X	Free online
Olff et al. (2021)	GPS	21 languages (not specified)^a^	Civilian	Comm	21 countries	7034	74				X		Free online
Bentley et al. (2014)	HTQ	Somali	Displ	Comm	United States	79	25.4	F & B	X		X		
Chukwuorji et al. (2017)	HTQ	Tiv	Displ	Comm	Nigeria	859	49.5	F & B	X	X	X		On request
Cohen et al. (2009)	HTQ	Kinyarwanda	Civilian	Comm	Rwanda	936	100	F & B	X		X		
De Fouchier et al. (2012)	HTQ	French	Displ	Clinical	England France	52	44	F & B			X	X	
Fawzi et al. (1997)	HTQ	Vietnamese	Displ	Comm	USA	74	Unclear			X	X		
Finkelstein (2016)	HTQ	Amharic	Civilian	Comm	Israel	478	46.1	F & B	X		X		
Hollander et al. (2007)	HTQ	Russian	PC/Vet	Clinical	Tajikistan	75	40	F & B	X			X	
Housen et al. (2018)	HTQ	Urdu	Civilian	Clinical	India	290	60	F & B	X		X	X	On request
Ichikawa et al. (2006)	HTQ	Dali	Displ	Comm	Japan	55	Unclear				X	X	
Kleijn et al. (2001)	HTQ	Farsi Serbo-Croation Russian	Displ	Clinical	Netherlands	S1: 64 S2:121 S3:62 S4:24	19				X		
Leaman and Gee (2012)	HTQ	French Amharic	Civilian	Comm	USA	131	54.9	F & B			X		
Lhewa et al. (2007)	HTQ	Tibetan	Displ	Clinical	USA	57	21.1	F & B			X	X	
Mollica et al. (1992)	HTQ	Khmer Lao Vietnamese	Displ	Comm	USA	91	62.6	F & B			X	X	
Mordeno et al. (2014)	HTQ	Filipino-Tagalog	Civilian	Comm	Philippines	737	62.8	F & B		X	X		
Schubert et al. (2016)	HTQ	Tetun	PC/Vet	Clinical	Timor-Leste	23	69.6	F & B			X		
Silove et al. (2014)	HTQ	Tetum	PC/Vet	Comm	Timor-Leste	1022	53.7	F & B	X			X	
Tay et al. (2017)	HTQ	Tetum	Civilian	Comm	Timor-Leste	2964	51	F & B	X		X		
Yeomans et al. (2008)	HTQ	Kirundi	Civilian	Comm	Burundi	78	36	F & B			X		
Yuval et al. (2021)	HTQ	Sudanese Arabic Tigrinya	Displ	Comm	Israel	148	0	F & B	X	X	X		
Zeligman et al. (2020)	HTQ	Setswana	Civilian	Comm	Botswana	300	53	F & B			X		
Renner et al. (2006)	HTQ IES-R	Chechnyan Farsi	Displ	Comm	Austria	150	S1: 50 S2: 22 S3: 8	F & B			X	X	
Patel et al. (2022)	HTQ-5	Hindi	Civilian	Comm	India	111	100	F & B	X		X	X	On request
Tay et al. (2017)	HTQ-5	Sinhalese	PC/Vet	Comm	Sri Lanka	4260	71.3	F & B	X	X			
Asukai et al. (2002)	IES-R	Japanese	Civilian	Mixed	Japan	1:487 2:73 3:86 4:658	1:9 2:52 3:60 4:44			X	X		
Brunet et al. (2003)	IES-R	French	Civilian	Comm	Canada	224	100	F & B		X	X	X	Free online
Caamaño et al. (2011)	IES-R	Spanish	Civilian	ED	Chile	278	53.60		X	X	X		
Gargurevich et al. (2009)	IES-R	Spanish	Civilian	Comm	Peru	S1:174 S2:562	S1:63.0 S2:59.0	F & B	X	X	X	X	
Ghezeljeh et al. (2013)	IES-R	Persian	Civilian	ED	Iran	55	45.5	F & B	X		X		
Grassi et al. (2021)	IES-R	Syrian Arabic	Displ	Comm	Turkey	288	57.2	F & B	X	X	X		Free online
Huang et al. (2006)	IES-R	Simplified Chinese	Civilian	Comm	Mainland China	439	100	F & B			X	X	
Iranmanesh et al. (2015)	IES-R	Persian	Civilian	Comm	Iran	200	50	F & B	X		X		
John & Russell (2007)	IES-R	Sinhalese	Civilian	Comm	Sri Lanka	30	Unclear	F & B		X	X	X	
King et al. (2009)	IES-R	Hebrew	Civilian	Mixed	Israel	235	45	F & B		X	X		
Klis et al. (2011)	IES-R	Mandinka & Wolof	Civilian	Clinical	Gambia	44	78.6				X		
Lim et al. (2009)	IES-R	Korean	Civilian	Clinical	South Korea	254	50	F & B		X	X	X	
Malinauskienė & Bernotaitė (2016)	IES-R	Lithuanian	Civilian	Comm	Lithuania	294	67	F & B		X	X		
Mystakidou et al. (2007)	IES-R	Greek	Civilian	Clinical	Greece	82	53.7	F & B		X	X		
Sharif Nia et al. (2021)	IES-R	Persian	Civilian	Comm	Iran	500	68.4	F & B	X	X	X		
Shin et al. (2009)	IES-R	Pashto	Civilian	Comm	Afghanistan	125	100	F & B	X		X		Free online
Tareen et al. (2012)	IES-R	Urdu	Civilian	Comm	Pakistan	118	unclear	F & B			X	X	On request
Warsini et al. (2015)	IES-R	Indonesian	Civilian	Comm	Indonesia	110	53.6	F & B	X	X	X		
Wu & Chan (2003)	IES-R	Traditional Chinese	Civilian	ED	HK SAR	116	44.8	F & B	X	X	X	X	
Choi et al. (2021)	ITQ	Korean	Civilian	Comm	Korea	251	19.5	F & B		X	X	X	Free online
Christen et al. (2021)	ITQ	German	Civilian	Comm	Germany	500	53	F & B		X	X	X	Free online
Donat et al. (2019)	ITQ	Brazilian Portuguese	Civilian	Mixed	Brazil	35	62.9	F & B	X		X		Free online
Gilbar et al. (2018)	ITQ	Hebrew	Civilian	Mixed	Israel	234	0	F & B		X	X		Free online
Hansen et al. (2021)	ITQ	Danish	Civilian	Clinical	Denmark	S1:40 S2: 1099	S1:47.5 S2:68.8	F & B		X	X	X	Free online
Hecker et al. (2018)	ITQ	Multiple	Displ	Comm	Switzerland	94	14.9	F & B			X		Free online
Ho et al. (2019)	ITQ	Chinese	Civilian	Comm	HK SAR	423	58.6	F & B	X	X	X		Free online
Ho et al. (2020)	ITQ	Japanese	Civilian	Comm	HK SAR Mainland China Taiwan Japan	1346	67.9	F & B		X	X	X	Free online
Hyland et al. (2018)	ITQ	Arabic	Displ	Clinical	Lebanon	110	80.2	F & B		X	X		Free online
Kazlauskas et al. (2018)	ITQ	Lithuanian	Civilian	Comm	Lithuania	280	77.5	F & B		X	X		Free online
Murphy et al. (2018)	ITQ	Luo	Civilian	Comm	Uganda	314	51	F & B		X	X		
Sele et al. (2020)	ITQ	Norwegian	Civilian	Clinical	Norway	202	84.7	F & B		X	X		Free online
Vallieres et al. (2018)	ITQ	Arabic	Displ	Comm	Lebanon	112	80.2	F & B	X	X	X		Free online
Vang et al. (2021)	ITQ	Danish	Mixed	Mixed	Denmark	S1:385 S2:147 S3:111 S4:178 S5:385	S1:85.6 S2:100 S3:41.4 S4:73.6 S5:48.5	F & B		X	X	X	Free online
Alhalal et al. (2017)	PCL	Arabic	Civilian	Clinical	Saudi Arabia	299	100		X	X	X		On request
Al-Turkait & Ohaeri (2014)	PCL	Arabic	Civilian	Comm	Kuwait	624	70.8	F & B			X		
Bahari et al. (2015)	PCL	Malay	Civilian	ED	Malaysia	63	20.6	F & B	X	X	X		
Blanc et al. (2016)	PCL	Haitian Creole	Civilian	Comm	Haiti	167	48.5	F & B			X		
Bonilla-Escobar et al. (2018)	PCL	Spanish	Civilian	Comm	Columbia	364	82.1				X		
Calbari & Anagnostopoulos (2010)	PCL	Greek	Civilian	Comm	Greece	312	60.2	F & B	X	X	X		
Carvalho et al. (2015)	PCL	Portuguese	PC/Vet	Comm	Portugal	86	0				X	X	
Carvalho et al. (2015)	PCL	Portuguese	PC/Vet	Clinical	Portugal	660	0	F & B		X	X		
Costa-Requena & Gil (2010)	PCL	Spanish	Civilian	Clinical	Spain	494	52			X	X		
Fernando (2008)	PCL	Sinhalese	Civilian	Comm	Sri Lanka	170	72	F & B			X		
Halcon et al. (2004)	PCL	Oromo Somali	Displ	Comm	USA	338	38.8	F & B			X		
Hem et al. (2012)	PCL	Norwegian	Civilian	Comm	Unclear	62	53	F & B				X	
Hinton et al. (2010)	PCL	Cambodian	Civilian	Clinical	Cambodia	220	62				X		
Hocker & Mehnert (2012)	PCL	German	Civilian	Comm	Germany	1594	67.9			X	X	X	
Marshall (2004)	PCL	Spanish	Civilian	Clinical	USA	120	6			X			
Mayer et al. (2020)	PCL	Tigrinya	Displ	Comm	Israel	18	100	F & B			X		
McDonald et al. (2019)	PCL	Somali	Displ	Comm	Kenya	250	57.2	F & B		X	X		
Orlando & Marshall (2002)	PCL	Spanish	Civilian	ED	USA	386	6	F & B		X	X		
Perera et al. (2013)	PCL	Somali Oromo	Displ	Comm	USA	437	48.74	F & B	X		X		
Regev & Slonim-Nevo (2019)	PCL	Arabic	Displ	Comm	Israel	330	19.7	F & B	X		X		On request
Semage et al. (2013)	PCL	Sinhalese	PC/Vet	Comm	Sri Lanka	1586	0	F & B	X	X	X	X	
Vera-Villarroel et al. (2011)	PCL	Spanish	Civilian	Comm	Chile	509	53	F & B		X	X	X	
Acarturk et al. (2021)	PCL-5	Arabic	Displ	Comm	Turkey	1678	52			X	X		
Boysan et al. (2017)	PCL-5	Turkish	Civilian	Mixed	Turkey	462	61				X	X	
Carvalho et al. (2020)	PCL-5	Portuguese	Civilian	Comm	Portugal	446	20	F & B	X	X	X		
Cheung et al. (2019)	PCL-5	Ukrainian Russian	Displ	Comm	Ukraine	2203	68.1				X		Free online
Hall et al. (2019)	PCL-5	Tagalog	Civilian	Comm	Macau	S1:130 S2: 99	S1: 100 S2:100	F & B	X		X	X	On request
Kruger-Gottschalk et al. (2017)	PCL-5	German	Civilian	Clinical	Germany	352	56.3	F & B		X	X	X	
Martínez-Levy et al. (2021)	PCL-5	Spanish	Civilian	Clinical	Mexico	68	76.5	F & B	X		X	X	
Mendoza et al. (2017)	PCL-5	Filipino	Civilian	Comm	China	261	100	F & B			X		
Mordeno et al. (2016)	PCL-5	Filipino-Tagalog	Displ	Comm	Philippines	460	72.8	F & B		X	X		
Patel et al. (2021)	PCL-5	Hindi	Civilian	Comm	India	112	100	F & B			X		
Sikkema et al. (2018)	PCL-5	Xhosa	Civilian	Clinical	South Africa	64	100	F & Bd			X		
Sveen et al. (2016)	PCL-5	Swedish	Civilian	Clinical	Sweden	62	70	F & B			X	X	
Verhey et al. (2018)	PCL-5	Shona	Civilian	Clinical	Zimbabwe	204	85.3	F & B			X	X	Free online
Zheng et al. (2020)	PCL-5	Chinese	Civilian	Comm	USA Mainland China HK SAR	558	79.2	F & B			X		
Yang et al. (2007)	PCL-C	Simplified Chinese	Civilian	Comm	Mainland China	186	unclear	F & B	X	X	X		
Fung et al. (2019)	PC-PTSD PCL-5	Chinese	Civilian	Clinical	Taiwan	56	66.1	F & B			X	X	On request
Jang et al. (2016)	PC-PTSD	Korean	Displ	Clinical	Korea	140	0	F & B	X		X	X	
Jung et al. (2018)	PC-PTSD	Korean	Civilian	Clinical	South Korea	252	50	F & B			X	X	
Akerblom et al. (2017)	PDS	Swedish	Civilian	Clinical	Sweden	463	72.1	F & B	X	X	X		
de Faria Cardoso et al. (2021)	PDS	Portuguese	Civilian	Clinical	Brazil	53	88.4	F & B	X		X	X	On request
Ertl et al. (2010)	PDS	Luo	PC/Vet	Comm	Uganda	504	56.9	F & B			X	X	
Griesel et al. (2006)	PDS	German	Civilian	Comm	Germany	143	60			X	X	X	
Hearn et al. (2012)	PDS	French	Civilian	Comm	Switzerland	287	63	F & B		X	X	X	
Hinton et al. (2018)	PDS	Vietnamese	Civilian	Comm	Vietnam	1004	56	F & B			X		
Myers et al. (2015)	PDS	Spanish	Civilian	Mixed	USA	S1:500	56.6	F & B		X			
Nickerson et al. (2015)	PDS	Multiple	Displ	Clinical	Switzerland	134	21.6	F & B			X		
Nickerson et al. (2019)	PDS	Arabic Farsi Tamil	Displ	Comm	Australia	1085	42.9	F & B	X		X		On request
Norris & Aroian (2008)	PDS	Arabic	Civilian	Comm	USA	453	100	F & B			X		
Odenwald et al. (2007)	PDS	Somali	PC/Vet	Comm	Somalia	135	1.5	F & B			X	X	
Selmo et al. (2019)	PDS	Arabic	Civilian	Clinical	Germany	1544	65.6	F & B		X			
Wyatt et al. (2017)	PDS	Local language of South Africa	Civilian	Clinical	South Africa	209	100	F & B			X		
Alghamdi & Hunt (2020)	PDS-5	Arabic	Civilian	Comm	Saudi Arabia	357	60	F & B	X	X	X		
Su et al. (2020)	PDS-5	Chinese	Mixed	Mixed	Taiwan	S1:138 S2:403 S3:181 S4:91 S5:90	S1:58.7 S2:65.8 S3:55.2 S4:44.0 S5:100	F & B		X	X	X	On request
Hecker et al. (2016)	PSS-I	Kishahili	Civilian	Comm	DRC	73	74				X		
Hinsberger et al. (2016)	PSS-I	Xhosa	Civilian	Comm	South Africa	290	0	F & B			X		
Specker & Nickerson (2022)	PSS-I	Arabic Farsi Tamil	Displ	Clinical	Australia	82	34.1	F & B			X		
Jaapar et al. (2014)	TSQ	Malay	Civilian	Clinical	Malaysia	50	28	F & B	X		X	X	On request
Rodriguez-Rey et al. (2016)	TSQ	Spanish	Civilian	Comm	Spain	S1:298 S2:189	S1:82.6 S2:84.1	F & B			X		Free online

^a^ At the moment of writing the GPS is available in 35 languages: https://www.global-psychotrauma.net/gps.

HTQ = Harvard Trauma Questionnaire; Comm = Community; Displ = Forcibly Displaced; DTS = Davidson Trauma Scale; ED = Emergency Department; F & B = Forward and Backward; HK SAR = Hong Kong Special Administrative Region; PC/Vet = Post-Conflict / Veteran; PCL = Posttraumatic Checklist; PCL-C = Posttraumatic Checklist-Civilian; IES-R = Impact of Events Scale-Revised; PCL-5 = Posttraumatic Checklist for the DSM-5; PDS = Posttraumatic Diagnostic Scale; PDS-5 =  Posttraumatic Diagnostic Scale-5 ITQ = International Trauma Questionnaire; GPS = Global Psychotrauma Screen; PSS-I = PTSD Symptom Scale-Interview; PC-PTSD = Primary Care PTSD Screen; TSQ = Trauma Screening Questionnaire; SPRINT = Short Post-Traumatic Stress Disorder Rating Interview; S(x) = Sample.

### 3.3. Translation, evaluations and availability

Eighty-three per cent (*n* = 105) of studies described using a forward and back translation procedure. Regarding the evaluation of translated questionnaires, 95.2% of studies (*n* = 120) used quantitative reliability analyses (e.g. internal consistency, test-retest reliability, inter-rater reliability, etc.), 47.6% (*n* = 60) used quantitative dimensionality evaluations (e.g. confirmatory factor analysis, bifactor modelling, measurement invariance *testing*), 38.9% (*n* = 49) used quantitative performance analyses (e.g. diagnostic accuracy, correlation with another screening questionnaire) and 33.3% (*n* = 42) used qualitative methods (e.g. pilot testing of the adapted questionnaire, qualitative interviews, face validity). Regarding the availability of the translated questionnaires, 17 (13.5%) questionnaires are freely available online, 5 (4.0%) were made freely available online by their authors after being contacted by the study team, and in 12 (9.5%) cases the authors stated that they are willing to share the questionnaires upon request. We were unable to obtain 92 (73.0%) of all questionnaires for different reasons (e.g. the authors declined or did not respond to our request). For a more detailed description of the results see Table 1 and the Supplementary Materials.

## 4. Discussion

The objective of this scoping review was to map the global availability of translations of evaluated PTSD screening questionnaires for adult populations. This was achieved by (1) identifying published, peer-reviewed screening questionnaires that were translated in at least two different languages, and (2) mapping these questionnaires across translation techniques, qualitative and quantitative evaluation procedures, and accessibility of translated questionnaires. This scoping review identified 12 PTSD screening questionnaires that had undergone at least two translations and evaluations from English into another language.

The breadth of the questionnaires identified in this review covers definitions of PTSD according to the DSM-IV, the DSM-5, and the ICD-11. While the 12 questionnaires identified in this review represent a broad sample of PTSD screening questionnaires, if this list is limited to current definitions (i.e. DSM-5 or ICD-11), many fewer questionnaires were identified (specifically HTQ-5, PDS-5, PCL-5, ITQ). The target languages included in this review highlight the diversity of translations and gaps to be addressed. The most frequent translations comprised targeted languages such as Arabic, Spanish, Chinese, German, Korean, and French. While these are dominant languages spoken in multiple countries, they tend to represent the dominant languages spoken in high income countries, and/or the language of the most dominant migrant groups that reside in those countries. The over-representation of high-income countries in target languages is reflected when viewing the geographic locations in which these studies were conducted. These include the USA, China, Germany, and Korea, while relatively fewer studies were conducted in parts of Africa, South America, Eastern Europe, Southeast Asia, and the Middle East. These findings are not surprising, given that most research funding comes from high income countries. However, for clinicians and researchers residing in low- and middle-income countries, the lack of available non-English screening questionnaires poses major challenges (e.g. when accurate PTSD screening is needed to make important decisions regarding triaging patients or allocating resources). While most of the world’s population resides in lower- and middle-income countries, mental health resources in these contexts are limited (Saxena et al., 2007). Amplifying this problem, is that only a small fraction (*n* = 34, 27.0%) of all identified questionnaires are freely available online or upon request from the developers. These inequities in research funding and resources may exacerbate the disparity in global mental health and mental health research. More global partnerships are needed to provide valid translations of PTSD screening questionnaires to reduce this gap.

The utility of translated PTSD screening questionnaires is highly dependent upon the quality of the translation. In this review, most studies reported using forward and blind-back translation procedures, according to best practice (Acquadro et al., 2008). However, the reporting on this process, such as the qualifications of the translators, how discrepancies were reconciled and/or the use of expert panels to review the final translation, varied substantially across different studies. In many instances, it was unclear if the translation process was conducted by the authors themselves, or was completed in an alternate study. Indeed, the lack of descriptions around translation processes precludes a more systematic review of the literature. Greater transparency and more standardized documentation of the translation process will be important to establish, as the utility of screeners is dependent upon linguistic accuracy. Equally important in the translation process is the evaluation of the translated questionnaire. The most frequently reported evaluation in this scoping review was quantitative reliability analyses. In most cases this was using Cronbach’s alpha, with only a few studies implanting methods such as test-retest analyses. Less than 40% of studies evaluated the diagnostic performance of the translated questionnaire. It is also worth noting that less than 34% of studies reported qualitative methods which includes basic practices such as investigating the face validity of the questionnaire. Given that direct translations may often not convey the appropriate information, utilizing and reporting this form of evaluation is crucial for ensuring that meaning is retained, rather than only relying on statistical methods. These results indicate that there was substantial variability regarding the reported evaluation procedures. It is worth noting that 132 studies were excluded during screening because they did not include any form of evaluation. Guidelines are needed on baseline evaluation requirements for translation use, in addition to more studies in which the primary aim is to evaluate the diagnostic utility of translated questionnaires.

While the translation and evaluation process of PTSD screening questionnaires can be costly and burdensome (Acquadro et al., 2008), this burden could be alleviated by making translations freely available for research and clinical use. For example, given that permission has been obtained from the authors of the original questionnaire, translated versions could be published alongside the article describing the translation and evaluation procedures. To disseminate the results from this review and increase access for clinicians and researchers, we created a website (https://tobiasrspiller.github.io/PTSD-Screener-Repo/) that provides global access to translated PTSD screening questionnaires. On this website, questionnaires can be downloaded or requested from the developers and the relevant studies are linked so that the developers can be credited appropriately. Moreover, the website also highlights gaps in the literature.

While this review aimed to map the availability of translated and evaluated PTSD screening questionnaires, some limitations should be noted. First, it was not the aim of this review to provide a quality assessment of each translated questionnaire. Information around translation is often lacking, precluding an assessment of this kind. Similarly, the review does not make recommendations on psychometric validity of the questionnaire. Rather, the aim was to present an overview in order to provide information for researchers and clinicians to make their own decisions. Second, to ensure feasibility of the review we planned to exclude all questionnaires that were translated only once. Following this preregistered protocol, we excluded the Short Post-Traumatic Stress Disorder Rating Interview (SPRINT), the only questionnaire for which we found less than two translations. This cut-off was partially arbitrary, as was the list of the included questionnaires, both not covering all existing questionnaires and translations. Future studies should aim to map these uncovered gaps, for example, questionnaires that were developed in a language other than English. Finally, this study only examined English questionaries that were translated into a target language. Therefore, there may have been non-English PTSD questionnaires that have been developed, which were outside the scope of this review.

To conclude, while the results identified 12 translated PTSD questionnaires, more transparency is needed around translation processes, and more rigorous evaluation methods are needed to ensure the utility of these measures in clinical and research contexts. Furthermore, more investment is needed in developing high quality translations of PTSD screening questionnaires in countries and language groups that have thus far been neglected. As the majority of existing translations were not accessible, more avenues for freely accessible translations of PTSD questionnaires are urgently needed. Making translations available in an online repository will hopefully help eliminate the need for duplicate translations and create space for more rigorous validation studies to be made widely available.

## Supplementary Material

Supplemental Material

Supplemental Material

Supplemental Material

Supplemental Material

Supplemental Material

Supplemental Material

Supplemental Material

## Data Availability

All relevant data is included in the supplement.
