# Supplementary material for: Mapping the availability of translated versions of posttraumatic stress disorder screening questionnaires for adults: A scoping review
Source: Eur J Psychotraumatol. 2022 Nov 25;13(2):2143019. doi: 10.1080/20008066.2022.2143019 (PMC9724641; doi:10.1080/20008066.2022.2143019)
Supplement: Supplemental Material [file ZEPT_A_2143019_SM9919.rtf]

---title: Create Figure 2 - showing number of studies per countrysubtitle: Version 1.0.0author:- <h5 style="font-style:italic"> Tobias R. Spiller^output:  html_document---```{r setup, include=FALSE}knitr::opts_chunk$set(echo = TRUE)```1. Relevant libraries are loaded.```{r Load Libraries, message=FALSE, warning=TRUE, include=FALSE}library(tmap)library(sf)library(tidyverse)library(readxl)```2. Extracted data and the map is loaded and combined```{r Load Data, echo=TRUE, message=FALSE, warning=TRUE}# Load Mapdata("World")# Load Extracted dataTranslations <- read_excel("Desktop/Data_Figure_2.xlsx",     col_types = c("text", "numeric"))# Combineworld2 <- left_join(World, Translations, by = "name")```3. Combined data is cleaned.```{r Cleaning, echo=TRUE, message=FALSE, warning=TRUE}# Data is adjusted manuallyworld2$Number_of_studies[169] <- 12 #USAworld2$Number_of_studies[58] <- 1 #UK# Antarctica is removedworld2 <- world2 %>%   filter(name != "Antarctica")```4. Figure is plotted```{r Plotting, echo=TRUE, message=FALSE, warning=TRUE}tmap_mode("plot")trans_map <- tm_shape(world2) +  tm_polygons("Number_of_studies",              breaks=c(1,3,6,8,12),              title='Number of studies',              labels = c("1 to 2", "3 to 5", "6 to 8", "8 to 12"))trans_map```4. Figure is saved```{r Save, echo=TRUE, message=FALSE, warning=TRUE}tmap_save(trans_map, "Desktop/Figure_2.pdf")```
