# Supplementary material for: Mapping the availability of translated versions of posttraumatic stress disorder screening questionnaires for adults: A scoping review
Source: Eur J Psychotraumatol. 2022 Nov 25;13(2):2143019. doi: 10.1080/20008066.2022.2143019 (PMC9724641; doi:10.1080/20008066.2022.2143019)
Supplement: Supplemental Material [file ZEPT_A_2143019_SM9918.docx]

**APPENDIX I**

**Questionnaires included in first review stage, listed alphabetically**

| **Name of the questionnaire** | **Abbreviation** | **Number of items** | **Reference** |
| --- | --- | --- | --- |
| Brief Trauma Questionnaire | BTQ | 10 | [1] |
| Davidson Trauma Scale | DTS | 17 | [2] |
| Global Psychotrauma Screen | GPS | 22 | [3, 4] |
| Harvard Trauma Questionnaire | HTQ | 30 | [5] |
| Harvard Trauma Questionnaire - 5 | HTQ-5 | 25 | [6] |
| Impact of Event Scale - Revised | IES-R | 22 | [7] |
| International Trauma Questionnaire | ITQ | 18 | [8] |
| Primary Care PTSD Screen | PC-PTSD | 4 | [9] |
| Primary Care PTSD Screen for DSM-5 | PC-PTSD-5 | 5 | [10] |
| PTSD Checklist | PCL | 17 | [11] |
| PTSD Checklist for DSM-5 | PCL-5 | 20 | [12] |
| Posttraumatic Diagnostic Scale | PDS | 17 | [13] |
| Posttraumatic Diagnostic Scale for DSM-5 | PDS-5 | 24 | [14] |
| PTSD Symptom Scale - Interview | PSS-I | 17 | [15] |
| PTSD Symptom Scale - Interview for DSM-5 | PSS-I-5 | 20 | [16] |
| SPAN Self-Report Screen | SPAN | 4 | [17] |
| Short Post-Traumatic Stress Disorder Rating Interview | SPRINT | 8 | [18] |
| Trauma Screening Questionnaire | TSQ | 10 | [19] |

**References**

1. Schnurr PP, Vielhauer M, Findler M. Brief Trauma Questionnaire. 1999.

2. Davidson JRT, Book SW, Colket JT, Tupler LA, Roth S, David D, et al. Assessment of a new self-rating scale for post-traumatic stress disorder. Psychol Med. 1997;27:153–60.

3. Olff M, Bakker A, Frewen P, Aakvaag H, Ajdukovic D, Brewer D, et al. Screening for consequences of trauma – an update on the global collaboration on traumatic stress. European Journal of Psychotraumatology. 2020;11:1752504.

4. Oe M, Kobayashi Y, Ishida T, Chiba H, Matsuoka M, Kakuma T, et al. Screening for psychotrauma related symptoms: Japanese translation and pilot testing of the Global Psychotrauma Screen. European Journal of Psychotraumatology. 2020;11:1810893.

5. Mollica RF, Caspi-Yavin Y, Bollini P, Truong T, Tor S, Lavelle J. The Harvard Trauma Questionnaire: Validating a cross-cultural instrument for measuring torture, trauma, and posttraumatic stress disorder in Indochinese refugees. Journal of Nervous and Mental Disease. 1992;180:111–6.

6. Berthold SM, Mollica RF, Silove D, Tay AK, Lavelle J, Lindert J. The HTQ-5: revision of the Harvard Trauma Questionnaire for measuring torture, trauma and DSM-5 PTSD symptoms in refugee populations. European Journal of Public Health. 2019;29:468–74.

7. Weiss DS, Marmar CR. The Impact of Event Scale - Revised. In: Wilson CJ, Keane TM, editors. Assessing psychological trauma and PTSD. New York: Guilford Publications; 1996. p. 399–411.

8. Cloitre M, Shevlin M, Brewin CR, Bisson JI, Roberts NP, Maercker A, et al. The International Trauma Questionnaire: development of a self-report measure of ICD-11 PTSD and complex PTSD. Acta Psychiatr Scand. 2018;138:536–46.

9. Prins A, Ouimette P, Kimerling R, Cameron RP, Hugelshofer DS, Shaw-Hegwer J, et al. The primary care PTSD screen (PC-PTSD): Development and operating characteristics. Primary Care Psychiatry. 2003;9:9–14.

10. Prins A, Bovin MJ, Smolenski DJ, Marx BP, Kimerling R, Jenkins-Guarnieri MA, et al. The Primary Care PTSD Screen for DSM-5 (PC-PTSD-5): Development and Evaluation Within a Veteran Primary Care Sample. J GEN INTERN MED. 2016;31:1206–11.

11. Blanchard EB, Jones-Alexander J, Buckley TC, Forneris CA. Psychometric properties of the PTSD checklist (PCL). Behaviour Research and Therapy. 1996;34:669–73.

12. Blevins CA, Weathers FW, Davis MT, Witte TK, Domino JL. The Posttraumatic Stress Disorder Checklist for *DSM-5* (PCL-5): Development and Initial Psychometric Evaluation. JOURNAL OF TRAUMATIC STRESS. 2015;28:489–98.

13. Foa EB, Cashman L, Jaycox L, Perry K. The validation of a self-report measure of posttraumatic stress disorder: the Posttraumatic Diagnostic Scale. Psychological assessment. 1997;9:445.

14. Foa EB, McLean CP, Zang Y, Zhong J, Powers MB, Kauffman BY, et al. Psychometric properties of the Posttraumatic Diagnostic Scale for DSM–5 (PDS–5). Psychological Assessment. 2016;28:1166–71.

15. Foa EB, Riggs DS, Dancu CV, Rothbaum BO. Reliability and validity of a brief instrument for assessing post‐traumatic stress disorder. Journal of traumatic stress. 1993;6:459–73.

16. Foa EB, McLean CP, Zang Y, Zhong J, Rauch S, Porter K, et al. Psychometric properties of the Posttraumatic Stress Disorder Symptom Scale Interview for DSM–5 (PSSI–5). Psychological Assessment. 2016;28:1159–65.

17. Meltzer-Brody S, Churchill E, Davidson JRT. Derivation of the SPAN, a brief diagnostic screening test for post-traumatic stress disorder. Psychiatry Research. 1999;88:63–70.

18. Connor KM, Davidson JRT. SPRINT: a brief global assessment of post-traumatic stress disorder: International Clinical Psychopharmacology. 2001;16:279–84.

19. Brewin CR, Rose S, Andrews B, Green J, Tata P, McEvedy C, et al. Brief screening instrument for post-traumatic stress disorder. The British Journal of Psychiatry. 2002;181:158–62.
