# Supplementary material for: Mapping the availability of translated versions of posttraumatic stress disorder screening questionnaires for adults: A scoping review
Source: Eur J Psychotraumatol. 2022 Nov 25;13(2):2143019. doi: 10.1080/20008066.2022.2143019 (PMC9724641; doi:10.1080/20008066.2022.2143019)
Supplement: Supplemental Material [file ZEPT_A_2143019_SM9914.docx]

**APPENDIX II**

To develop the search strategy, keywords for each concept outlined in the inclusion criteria were searched.

| Concept | Keywords |
| --- | --- |
| Posttraumatic Stress Disorder | Posttraumatic Stress Disorder OR PTSD |
| Screening Questionnaire | Table 2 (OR) |
| Translation & Cultural Adaptation | Transl* OR cultur* OR cross-cultural |

**Table 1**

**Table 2**

| **Name of the questionnaire** | **Abbreviation** |
| --- | --- |
| Brief Trauma Questionnaire | BTQ |
| Davidson Trauma Scale | DTS |
| Global Psychotrauma Screen | GPS |
| Harvard Trauma Questionnaire | HTQ |
| Harvard Trauma Questionnaire - 5 | HTQ-5 |
| Impact of Event Scale - Revised | IES-R |
| International Trauma Questionnaire | ITQ |
| Primary Care PTSD Screen | PC-PTSD |
| Primary Care PTSD Screen for DSM-5 | PC-PTSD-5 |
| PTSD Checklist | PCL |
| PTSD Checklist for DSM-5 | PCL-5 |
| Posttraumatic Diagnostic Scale | PDS |
| Posttraumatic Diagnostic Scale for DSM-5 | PDS-5 |
| PTSD Symptom Scale - Interview | PSS-I |
| PTSD Symptom Scale - Interview for DSM-5 | PSS-I-5 |
| SPAN Self-Report Screen | SPAN |
| Short Post-Traumatic Stress Disorder Rating Interview | SPRINT |
| Trauma Screening Questionnaire | TSQ |

The screening questionnaires included into the review are outlined in Table 2. They were all added as separate keywords into the search queries. The final search queries were built as follows:

1. The keywords were nested on the concept level by the Boolean operator “OR”.
2. The keywords of each level were joint by Boolean operator “AND”.

**1. Embase**

Brief Trauma Questionnaire .mp. OR

Davidson Trauma Scale.mp. OR

Global Psychotrauma Screen.mp. OR

Harvard Trauma Questionnaire.mp. OR

exp "Impact of Events Scale"/ OR

International Trauma Questionnaire.mp. OR

Primary Care PTSD Screen.mp. OR

PTSD Checklist.mp. OR

Posttraumatic Diagnostic Scale.mp. OR

PTSD symptom scale interview.mp. OR

Short Post-Traumatic Stress Disorder Rating Interview.mp. OR

Trauma Screening Questionnaire.mp.

AND

exp posttraumatic stress disorder/ or (ptsd or posttraumatic stress or post traumatic stress or trauma or traumatic).mp.

AND

(transl*.mp. OR cult*.mp.)

**2. MEDLINE**

Brief Trauma Questionnaire[Text Word] OR

Davidson Trauma Scale[Text Word] OR

Global Psychotrauma Screen[Text Word] OR

Harvard Trauma Questionnaire[Text Word] OR

Impact of event scale revised[Text Word] OR

International Trauma Questionnaire[Text Word] OR

Primary Care PTSD Screen[Text Word] OR

PTSD Checklist[Text Word] OR

Posttraumatic Diagnostic Scale[Text Word] OR

PTSD symptom scale interview[Text Word] OR

Short Post-Traumatic Stress Disorder Rating Interview[Text Word] OR

Trauma Screening Questionnaire[Text Word]

AND

(PTSD OR post traumatic OR posttraumatic OR Stress Disorders, Post-Traumatic[Mesh] OR trauma OR traumatic)

AND

(transl*[Text Word] OR Translations[Mesh] OR cross-cultural[Text Word] OR cultur*[Text Word] OR Cross-Cultural Comparison[Mesh])

*OVID MEDLINER*

Brief Trauma Questionnaire .mp. OR

Davidson Trauma Scale.mp. OR

Global Psychotrauma Screen.mp. OR

Harvard Trauma Questionnaire.mp. OR

Impact of Events Scale.mp. OR

International Trauma Questionnaire.mp. OR

Primary Care PTSD Screen.mp. OR

PTSD Checklist.mp. OR

Posttraumatic Diagnostic Scale.mp. OR

PTSD symptom scale interview.mp. OR

Short Post-Traumatic Stress Disorder Rating Interview.mp. OR

Trauma Screening Questionnaire.mp.

AND

exp posttraumatic stress disorder/

AND

(transl*.mp. OR cult*.mp. OR exp Cross-Cultural Comparison/)

**3. PsycInfo**

Brief Trauma Questionnaire.mp. OR

Davidson Trauma Scale.mp. OR

Global Psychotrauma Screen.mp. OR

Harvard Trauma Questionnaire.mp. OR

Impact of Events Scale.mp. OR

International Trauma Questionnaire.mp. OR

Primary Care PTSD Screen.mp. OR

PTSD Checklist.mp. OR

Posttraumatic Diagnostic Scale.mp. OR

PTSD symptom scale interview.mp. OR

Short Post-Traumatic Stress Disorder Rating Interview.mp. OR

Trauma Screening Questionnaire.mp.

AND

exp posttraumatic stress disorder/(ptsd or posttraumatic stress or post traumatic stress or trauma or traumatic).mp.

AND

(exp Foreign Language Translation/ OR transl*.mp. OR cult*.mp.)
