# Supplementary material for: Mapping the availability of translated versions of posttraumatic stress disorder screening questionnaires for adults: A scoping review
Source: Eur J Psychotraumatol. 2022 Nov 25;13(2):2143019. doi: 10.1080/20008066.2022.2143019 (PMC9724641; doi:10.1080/20008066.2022.2143019)
Supplement: Supplemental Material [file ZEPT_A_2143019_SM9913.docx]

**APPENDIX III**

**Inclusion and exclusion criteria list Stage - 1**

| **Inclusion** |
| --- |
| Study evaluated one of the PTSD Screening questionnaires listed in Appendix I |
| Translation *and* quantitative or qualitative validation of one of the questionnaires listed in Appendix I to a non-English speaking population. |
| Article written in either English, German, Spanish, French, Chinese, Filipino and Hebrew. |

| **Exclusion** |
| --- |
| Not peer-reviewed |
| Non-adult population (population included individuals under 18 years of age) |
