# Supplementary material for: Mapping the availability of translated versions of posttraumatic stress disorder screening questionnaires for adults: A scoping review
Source: Eur J Psychotraumatol. 2022 Nov 25;13(2):2143019. doi: 10.1080/20008066.2022.2143019 (PMC9724641; doi:10.1080/20008066.2022.2143019)
Supplement: Supplemental Material [file ZEPT_A_2143019_SM9908.docx]

Supplementary Table 1. Evaluation Description of Translated Questionnaires

| **Authors** | **Questionnaire** | **Target language** | **Translation** | **Translation - description** | **Qualitative** | **Qualitative - description** | **Dimensionality** | **Dimensionality - description** | **Reliability** | **Reliability - description** | **Performance** | **Performance - description** |
| --- | --- | --- | --- | --- | --- | --- | --- | --- | --- | --- | --- | --- |
| Acarturk 2021 | PCL-5 | Arabic |  |  |  |  | X | CFA | X | Consistency (Cronbach) & Rho |  |  |
| Akerblom 2017 | PDS | Swedish | Forward & Backward | Panel/Team | X |  | X | CFA | X | Consistency (Cronbach) |  |  |
| Alghamdi 2020 | PDS-5 | Arabic | Forward & Backward |  | X | Interviews | X | CFA | X | Consistency (Cronbach) & test-retest |  |  |
| Alhalal 2017 | PCL | Arabic |  |  | X | Pilot | X | CFA | X | Consistency (Cronbach) |  |  |
| Al-Turkait 2014 | PCL | Arabic | Forward & Backward |  |  |  |  |  | X | Consistency (Cronbach) |  |  |
| Asukai 2002 | IES-R | Japanese |  |  |  |  | X | Cluster analysis | X | Consistency (Cronbach) & test-retest |  |  |
| Bahari 2015 | PCL | Malay | Forward & Backward | Panel/Team | X | Face validity Pilot | X | EFA | X | Consistency (Cronbach) & test-retest |  |  |
| Bentley 2014 | HTQ | Somali | Forward & Backward |  | X | Panel/Team |  |  | X | Consistency (Cronbach) |  |  |
| Blanc 2016 | PCL | Haitian Creole | Forward & Backward |  |  |  |  |  | X | Consistency (Cronbach) |  |  |
| Bobes et al., (2000) | DTS | Spanish | Forward & Backward |  |  |  |  |  | X | Consistency (Cronbach) & test-retest | X | Sens Spec ROC |
| Bonilla-Escobar 2018 | PCL | Spanish |  |  |  |  |  |  | X | Consistency (Cronbach) |  |  |
| Boysan 2017 | PCL-5 | Turkish |  |  |  |  |  | CFA | X | Composite reliability | X | Correlations with non-PTSD scales for Convergent validity ROC curve for optimal cut-off scores specificity and sensitivity. |
| Brunet 2003 | IES-R | French | Forward & Backward |  |  |  | X | PCA | X | Consistency (Cronbach) | X | Convergent Validity Correlation with other questionnaire |
| Calbari 2010 | PCL | Greek | Forward & Backward |  | X | Pilot | X | EFA | X | Consistency (Cronbach) |  |  |
| Camano et al. (2011) | IES-R | Spanish |  |  |  |  |  |  | X | Consistency (Cronbach) & test-retest | X | Correlation with other questionnaires, Diagnostic accuracy |
| Carvalho 2015 | PCL | Portuguese | Forward & Backward |  |  |  | X | CFA | X | Consistency (Cronbach) & test-retest |  |  |
| Carvalho 2015 | PCL | Portuguese |  |  |  |  |  |  | X | Consistency (Cronbach) | X | ROC Kappa |
| Carvalho 2020 | PCL-5 | Portuguese | Forward & Backward |  | X | Pilot | X | CFA | X | Consistency (Cronbach) & test-retest |  |  |
| Chen 2001 | DTS | Chinese | Forward & Backward |  |  |  | X | PCA | X | Consistency (Cronbach) & test-retest | X | Concurrent validity ROC |
| Cheung 2019 | PCL-5 | Ukrainian Russian |  |  |  |  |  |  | X | Consistency (Cronbach) |  |  |
| Choi 2021 | ITQ | Korean | Forward & Backward |  |  |  | X | CFA | X | Consistency (Cronbach) | X | Discriminant validity between CPTSD & PTSD and non-diagnosed group across |
| Christen 2021 | ITQ | German | Forward & Backward |  |  |  | X | CFA  PCA | X | IRT | X | IRT |
| Chukwuorji 2017 | HTQ | Tiv | Forward & Backward |  | X | Conceptual fidelity | X | Factor | X | Consistency (Cronbach) |  |  |
| Cohen 2009 | HTQ | Kinyarwanda | Forward & Backward | Panel/Team | X | Pilot |  |  | X | Consistency (Cronbach) |  |  |
| Costa-Requena 2010 | PCL | Spanish |  |  |  |  | X | EFA | X | Consistency (Cronbach) |  |  |
| deFariaCardoso 2021 | PDS | Portuguese | Forward & Backward |  | X | Pilot |  |  | X | Consistency (Cronbach) | X | Short-version-PDS scale correlated positively with the DSM5 scale  ROC |
| deFouchier 2012 | HTQ | French | Forward & Backward | Brislins method |  |  |  |  | X | Consistency (Cronbach) | X | Criterion validity compared with SCID |
| Donat 2019 | ITQ | Brazilian Portuguese | Forward & Backward | Panel/Team | X | Comprehension |  |  | X | Interrater Reliability |  |  |
| Ertl 2010 | PDS | Luo | Forward & Backward |  |  |  |  |  | X | Consistency (Cronbach) | X | ROC with CAPS-5 |
| Fawzi 1997 | HTQ | Vietnamese |  |  |  |  | X | PCA | X | Consistency (Cronbach) |  |  |
| Fernando 2008 | PCL | Sinhalese | Forward & Backward |  |  | Pilot |  |  | X | Consistency (Cronbach) |  |  |
| Finkelstein 2016 | HTQ | Amharic | Forward & Backward |  | X | Pilot |  |  | X | Consistency (Cronbach) |  |  |
| Fung 2019 | PC-PTSD PCL-5 | Chinese | Forward & Backward |  |  |  |  |  | X | Consistency (Cronbach) | X | Correlated with PC-PTD-5 Sensitivity and Specificity |
| Gargurevich 2009 | IES-R | Spanish | Forward & Backward |  | X | Panel/Team | X | CFA | X | Consistency (Cronbach) | X | Discriminant validity with two samples & Concurrent validity with CAPS |
| Ghezeljeh 2013 | IES-R | Persian | Forward & Backward | Panel/Team | X | Face validity Content Validity Pilot |  |  | X | Consistency (Cronbach) |  |  |
| Gilbar 2018 | ITQ | Hebrew | Forward & Backward |  |  |  | X | CFA | X | Consistency (tau) |  |  |
| Grassi 2021 | IES-R | Syrian Arabic | Forward & Backward |  | X | Panel/Team | X | CFA Measurement invariance | X | Consistency (Cronbach) |  |  |
| Griesel 2006 | PDS | German |  |  |  |  | X | EFA | X | Consistency (Cronbach) & test-retest | X | Specificity and Sensitivity with SCID-I & CAPS correlation with questionnaire PTSD |
| Halcon 2004 | PCL | Oromo Somali | Forward & Backward |  |  |  |  |  | X | Consistency (Cronbach) |  |  |
| Hall 2019 | PCL-5 | Tagalog | Forward & Backward | Panel/Team | X | Pilot |  |  | X | Consistency (Cronbach) & test-retest | X | Specificity and Sensitivity with MINI |
| Hansen 2021 | ITQ | Danish | Forward & Backward | Panel/Team |  |  | X | CFA | X | Consistency (Cronbach) | X | Diagnostic accuracy compared with Clinical diagnostic interview & Concurrent discriminant validity with PCL-5 |
| Hearn 2012 | PDS | French | Forward & Backward |  |  |  | X | CFA | X | Consistency (Cronbach) | X | Correlations with IESR |
| Hecker 2016 | PSS-I | Kishahili |  | Panel/Team |  |  |  |  | X | Consistency (Cronbach) |  |  |
| Hecker 2018 | ITQ | French German Spanish Arabic Turkish Tamil | Forward & Backward |  |  |  |  |  | X | Consistency (Cronbach) |  |  |
| Hem 2012 | PCL | Norwegian | Forward & Backward |  |  |  |  |  |  |  | X | SCID Sensitivity and Specificity |
| Hinsberger 2016 | PSS-I | Xhosa | Forward & Backward | Panel/Team |  |  |  |  | X | Consistency (Cronbach) Interrater Reliability |  |  |
| Hinton 2010 | PCL | Cambodian |  |  |  |  |  |  | X | Interrater Reliability Test-retest |  |  |
| Hinton 2018 | PDS | Vietnamese | Forward & Backward |  |  |  |  |  | X | Consistency (Cronbach) (unclear) |  |  |
| Ho 2019 | ITQ | Chinese | Forward & Backward |  | X | Face validity Content Validity Index Pilot | X | CFA | X | Consistency (Cronbach) & test-retest |  |  |
| Ho 2020 | ITQ | Japanese | Forward & Backward |  |  |  | X | CFA | X | Consistency (Cronbach) | X | SEM with other constructs |
| Hocker 2012 | PCL | German |  |  |  |  | X | EFA & CFA | X | Consistency (Cronbach) | X | Correlation IES-R ROC |
| Hollander 2007 | HTQ | Russian | Forward & Backward |  | X | Pilot |  |  |  |  | X | Compared with structured interviews |
| Housen 2018 | HTQ | Kashmiri | Forward & Backward |  | X | Pilot |  |  | X | Consistency (Cronbach) | X | Senstivity Specificity analysis compared with MINI |
| Huang et al., (1992) | IES-R | Simplified Chinese |  |  | X | Pilot | X | EFA | X | Temporal stability |  |  |
| Hyland 2018 | ITQ | Arabic | Forward & Backward |  |  |  | X | LCA | X | Consistency (Cronbach) |  |  |
| Ichikawa 2006 | HTQ | Dali |  |  |  |  |  |  | X | Consistency (Cronbach) | X | score vs algorithm |
| Iranmanesh 2015 | IES-R | Persian | Forward & Backward | Panel/Team | X | Pilot |  |  | X | Consistency (Cronbach) |  |  |
| Jaapar 2014 | TSQ | Malay | Forward & Backward |  | X | Face validity Pilot |  |  | X | Consistency (Cronbach) | X | Correlation with CAPS & Sensitivity and Specificity |
| Jang 2016 | PC-PTSD | Korean | Forward & Backward |  | X | Pilot |  |  | X | Consistency (Cronbach) & test-retest | X | Diagnostic accuracy Correlation with SCID ROC analysis |
| John 2007 | IES-R | Sinhalese | Forward & Backward |  |  |  | X | PCA | X | Consistency (Cronbach) & Interrater Reliability | X | ROC analysis |
| Jung 2018 | PC-PTSD | Korean | Forward & Backward |  |  |  |  |  | X | Consistency (Cronbach) & test-retest | X | ROC analysis |
| Karanikola 2021 | DTS | Greek-Cypriot | Forward & Backward | Panel/Team | X | Face validity | X | CFA | X | Consistency (Cronbach) & test-retest | X |  |
| Kazlauskas 2018 | ITQ | Lithuanian | Forward & Backward |  |  |  | X | CFA  LCA | X | Consistency (Cronbach) |  |  |
| King 2009 | IES-R | Hebrew | Forward & Backward |  |  |  | X | Measurement invariance | X | Consistency (Cronbach) |  |  |
| Kleijn 2001 | HTQ | Farsi Serbo-Croation Russian |  |  |  |  |  |  | X | Consistency (Cronbach) |  |  |
| Klis 2011 | IES-R | Mandinka Wolof |  |  |  |  |  |  | X | Consistency (Cronbach) |  |  |
| Kontoangelos 2017 | DTS | Greek | Forward & Backward |  |  |  | X | PCA | X | Consistency (Cronbach) & test-retest |  |  |
| Kruger-Gottschalk 2017 | PCL-5 | German | Forward & Backward |  |  |  | X | CFA | X | Consistency (Cronbach) & test-retest | X | Correlation with CAPS-5 ROC |
| Leaman 2012 | HTQ | French Amharic | Forward & Backward |  |  |  |  |  | X | Consistency (Cronbach) |  |  |
| Leiva-Bianchi 2013 | DTS | Spanish |  |  |  |  | X | EFA  CFA | X | Consistency (Cronbach) | X | Correlation DTS |
| Lhewa 2007 | HTQ | Tibetan | Forward & Backward |  |  |  |  |  | X | Consistency (Cronbach) | X | ROC analysis SCID |
| Lim 2009 | IES-R | Korean | Forward & Backward |  |  |  | X | PCA | X | Consistency (Cronbach) & test-retest | X | Correlation with CAPS & ROC |
| Malinauskienƒó 2016 | IES-R | Lithuanian | Forward & Backward |  |  |  | X | PCA | X | Consistency (Cronbach) |  |  |
| Marshall 2004 | PCL | Spanish |  |  |  |  | X | CFA Measurment invariance |  |  |  |  |
| Martinez-Levy 2021 | PCL-5 | Spanish | Forward & Backward |  | X | Pilot |  |  | X | Consistency (Cronbach) | X | ROC with CAPS-5 |
| Mayer 2020 | PCL | Tigrinya | Forward & Backward |  |  |  |  |  | X | Consistency (Cronbach) |  |  |
| McDonald 2019 | PCL | Somali | Forward & Backward |  |  |  | X | Dimensionality analysis IRT | X | Rasch analysis |  |  |
| Mendoza 2017 | PCL-5 | Filipino | Forward & Backward |  |  |  |  |  | X | Consistency (Cronbach) |  |  |
| Miranda (2006) | DTS | Spanish |  |  |  |  | X | EFA  CFA | X | Consistency (Cronbach) |  |  |
| Mollica 1992 | HTQ | Khmer Lao Vietnamese | Forward & Backward |  |  |  |  |  | X | Consistency (Cronbach) & Interrater Reliability & test-retest | X | Sensitivity/Specificity  Correlation with other PTSD questionnaire |
| Mordeno 2014 | HTQ | Filipino-Tagalog | Forward & Backward | Panel/Team |  |  | X | CFA | X | Consistency (Cronbach) |  |  |
| Mordeno 2016 | PCL-5 | Filipino-Tagalog | Forward & Backward |  |  |  | X | CFA | X | Consistency (rho) |  |  |
| Murphy 2018 | ITQ | Luo | Forward & Backward |  |  |  | X | CFA | X | Consistency (Cronbach) |  |  |
| Myers 2015 | PDS | Spanish | Forward & Backward |  |  |  | X |  |  | Consistency (Cronbach) |  |  |
| Mystakidou 2007 | IES-R | Greek | Forward & Backward |  |  |  | X | CFA | X | Consistency (Cronbach) & test-retest |  |  |
| Nickerson 2015 | PDS | German English Turkish Arabic Farsi Tamil | Forward & Backward |  |  |  |  |  | X | Consistency (Cronbach) |  |  |
| Nickerson 2019 | PDS | Arabic Farsi Tamil | Forward & Backward |  | X | Pilot |  |  | X | Consistency (Cronbach) |  |  |
| Norris 2008 | PDS | Arabic | Forward & Backward |  |  |  |  |  | X | Consistency (Cronbach) & test-retest |  |  |
| Odenwald 2007 | PDS | Somali | Forward & Backward |  |  |  |  |  | X | Consistency (Cronbach) | X | ROC CIDI |
| Oe 2020 | GPS | Japanese | Forward & Backward |  | X | Pilot |  |  | X | Consistency (Cronbach) | X | Correlation PCL-5 |
| Olff 2020 | GPS | Arabic German Indonesian | Forward & Backward |  | X | Pilot |  |  | X | Consistency (Cronbach) | X | Correlation CAPS, PCL-5 |
| Olff 2021 | GPS | 21 languages |  |  |  |  |  |  | X | Consistency (Cronbach) |  |  |
| Orlando 2002 | PCL | Spanish | Forward & Backward |  |  |  | X | CFA | X | Results of differential item functioning |  |  |
| Patel 2021 | PCL-5 | Hindi | Forward & Backward |  |  |  |  |  | X | Consistency (Cronbach) |  |  |
| Patel 2022 | HTQ  PCL-5 | Hindu | Forward & Backward |  | X | Comprehension  Pilot |  |  | X | Consistency (Cronbach) & Interrater Reliability | X | Correlation with PCL-5 HTQ5 |
| Perera 2013 | PCL | Somali Oromo | Forward & Backward |  | X | Pilot |  |  | X | Consistency (Cronbach) |  |  |
| Regev 2019 | PCL | Arabic | Forward & Backward |  | X | Pilot |  |  | X | test-retest |  |  |
| Renner 2006 | HTQ   IES-R | Chechnyan  Farsi | Forward & Backward |  |  |  |  |  | X | Consistency (Cronbach) | X | ROC CAPS |
| Rodriguez-Rey 2019 | TSQ | Spanish | Forward & Backward |  |  |  |  |  | X | Consistency (Cronbach) |  |  |
| Schubert 2016 | HTQ | Tetun | Forward & Backward |  |  |  |  |  | X | Consistency (Cronbach) |  |  |
| Sele 2020 | ITQ | Norwegian | Forward & Backward |  |  |  | X | CFA | X | G-Theory G-Study D-Study |  |  |
| Selmo 2019 | PDS | Arabic | Forward & Backward |  |  |  | X | CFA |  |  |  |  |
| Semage 2013 | PCL | Sinhalese | Forward & Backward | Panel/Team | X | Pilot | X | CFA | X | Consistency (Cronbach) | X | ROC Clinical |
| Seo 2008 | DTS | Korean | Forward & Backward |  |  |  | X | CFA | X | Consistency (Cronbach) & test-retest | X | Correlation with CAPS & ROC MINI |
| SharifNia 2021 | IES-R | Persian | Forward & Backward | Panel/Team | X | Pilot | X | EFA  CFA | X | Consistency (Cronbach) & McDonald CR |  |  |
| Shin 2009 | IES-R | Pashto | Forward & Backward |  | X | Pilot |  |  | X | Consistency (Cronbach) |  |  |
| Sikkema 2018 | PCL-5 | Xhosa | Forward & Backward |  |  |  |  |  | X | Consistency (Cronbach) |  |  |
| Silove 2014 | HTQ | Tetum | Forward & Backward |  | X | Pilot |  |  |  |  | X | Diagnostic accuracy compared with SCID |
| Specker 2022 | PSS-I | Arabic Farsi Tamil | Forward & Backward |  |  |  |  |  | X | Consistency (Cronbach) |  |  |
| Su 2020 | PDS-5 | Chinese | Forward & Backward |  |  |  | X | CFA | X | Consistency (Cronbach) & test-retest | X | Correlation with PDS5 |
| Sveen 2016 | PCL-5 | Swedish | Forward & Backward |  |  |  |  |  | X | Consistency (Cronbach) | X | Correlation with IES-R |
| Tareen 2012 | IES-R | Urdu | Forward & Backward |  |  |  |  |  | X | Consistency (Cronbach) | X |  |
| Tay 2017 | HTQ | Tetum | Forward & Backward |  | X | Pilot |  |  | X | Consistency (Cronbach) |  |  |
| Tay 2017 | HTQ | Sinhalese | Forward & Backward |  | X | Pilot | X | CFA |  |  |  |  |
| Vallieres 2018 | ITQ | Arabic | Forward & Backward |  | X | Interviews | X | CFA | X | Composite reliability |  |  |
| Vang 2021 | ITQ | Danish | Forward & Backward |  |  |  | X | CFA | X | Consistency (Cronbach) | X |  |
| Vera-Villarroel 2011 | PCL | Spanish | Forward & Backward |  |  |  | X | CFA | X | Consistency (Cronbach) | X |  |
| Verhey 2018 | PCL-5 | Shona | Forward & Backward | Panel/Team |  |  |  |  | X | Consistency (Cronbach) | X | ROC CAPS |
| Warsini 2015 | IES-R | Indonesian | Forward & Backward | Panel/Team | X | Pilot | X | CFA | X | Consistency (Cronbach) & test-retest |  |  |
| Wu 2003 | IES-R | Chinese | Forward & Backward |  |  |  | X | PCA | X | Consistency (Cronbach) |  |  |
| Wyatt 2017 | PDS | Local language of South Africa | Forward & Backward |  |  |  |  |  | X | Consistency (Cronbach) |  |  |
| Xiaoyun et al., (2007) | PCL-C | Simplified Chinese | Forward & Backward |  | X | Face validity | X | PCA | X | Consistency (Cronbach) & test-retest | X |  |
| Yeomans 2008 | HTQ | Kirundi | Forward & Backward |  |  |  |  |  | X | Consistency (Cronbach) |  |  |
| Yuval 2021 | HTQ | Sudanese Arabic Tigrinya | Forward & Backward |  | X | Pilot | X | Network estimation | X | Consistency (Cronbach) |  |  |
| Zeligman 2020 | HTQ | Setswana | Forward & Backward |  |  |  |  |  | X | Consistency (Cronbach) |  |  |
| Zheng 2020 | PCL-5 | Chinese | Forward & Backward |  |  |  |  |  | X | Consistency (Cronbach) |  |  |
|  |  |  |  |  |  |  |  |  |  |  |  |  |

Note. HTQ=Harvard Trauma Questionnaire; DTS=Davidson Trauma Scale; PCL=Posttraumatic Checklist; PCL-C=Posttraumatic Checklist-Civilian; IES-R=Impact of Events Scale-Revised; PCL-5=Posttraumatic Checklist for the DSM-5; PDS= Posttraumatic Diagnostic Scale; PDS-5= Posttraumatic Diagnostic Scale-5 ITQ=International Trauma Questionnaire; GPS=Global Psychotrauma Screen; PSS-I= PTSD Symptom Scale-Interview; PC-PTSD=Primary Care PTSD Screen; TSQ=Trauma Screening Questionnaire; SPRINT=Short Post-Traumatic Stress Disorder Rating Interview; S(n):=Sample
